# Optimising the design and evaluation of pilot work to inform the main trial: a review of current evidence and consideration of future practices

**DOI:** 10.1186/1745-6215-14-S1-O17

**Published:** 2013-11-29

**Authors:** Elaine O'Connell Francischetto, Kerry Avery, Chris Metcalfe, Paula Williamson, Carrol Gamble, Jane Blazeby

**Affiliations:** 1 University of Bristol, Bristol, UK; 2 University of Liverpool, Liverpool, UK

## Background

Pilot work may inform the feasibility and design of randomised controlled trials (RCTs), but consensus regarding terminology and selection of appropriate pilot work to inform a main trial design is lacking. This ongoing work reviews definitions of pilot work and examines best methods for determining successful progression to a main trial.

## Methods

PubMed was searched for articles with ‘pilot studies' in the title (inception-03/04/2013). Relevant papers and reference lists were reviewed to identify and refine definitions of different types of pilot work. Definitions informed structured searches of the top 10 medical journals and the journal ‘Trials' to identify different types of pilot work and how, if done well, they might inform the design and progression to a main trial.

## Results

Different terms were often used to describe similar types of pilot work (31 terms identified from 289 publications). Some terms (n=19,61%) lacked definitions, whereas others had multiple definitions (n=12,39%). Definitions were refined following discussion between reviewers. Pilot work can inform a RCT in numerous ways, including recalculating sample sizes and assessing study timelines and processes, but there is wide variation, potential bias and no standard practices. Progression criteria may inform guidance on when and how pilot work can continue to a main trial.

## Conclusion

Recommendations for defining pilot work to improve consensus and maximise its value will be presented. Guidance for assessing pilot work success, including stop/go/amend criteria and the role of the Data Monitoring Committee, will be developed to inform decisions about progression to a main RCT.

